# Genetic factors may load the gun, but environmental factors pull the trigger: MedDiet and DII in rheumatoid arthritis

**DOI:** 10.3389/fnut.2025.1629896

**Published:** 2025-09-17

**Authors:** Büşra Açıkalın Göktürk, Nevin Sanlier

**Affiliations:** ^1^Department of Gastronomy and Culinary Arts, Faculty of Fine Arts, Design and Architecture, Ankara Medipol University, Ankara, Türkiye; ^2^Department of Nutrition and Dietetics, School of Health Sciences, Ankara Medipol University, Ankara, Türkiye

**Keywords:** Rheumatoid arthritis, Mediterranean diet, anti-inflammatory, diet pattern, DIET pathway

## Abstract

Rheumatoid arthritis (RA) is a chronic inflammatory autoimmune disease that causes progressive joint destruction. It affects ~1% of the world population and is more common in women aged 20–45 years than in men. RA causes involvement of small joints such as the joints of the hands and feet, pain, swelling, stiffness and loss of function in the joints. In recent years, Mediterranean diet and anti-inflammatory diet models have attracted attention in the medical nutrition therapy of patients with RA. These dietary patterns have been suggested to reduce disease risk and complications and improve disease manifestations. Mediterranean and inflammatory dietary patterns contain antioxidant vitamins and minerals, omega-3, polyunsaturated fatty acids and may have effects on inflammation and pain. In addition, dietary patterns may be effective in preventing free radical formation and increased cytokine levels due to their antioxidant and anti-inflammatory properties. In addition, by decreasing inflammatory markers and increasing antioxidant mechanism, it may be effective in reducing the level of disease activity, clinical and biochemical findings and increasing the quality of life. While the Mediterranean Diet and diet inflammatory index (DII) show promise in managing RA, it is important to consider the variability of individual responses to dietary interventions. There are conflicting results regarding the efficacy of the MedDiet in reducing disease activity and further research is needed to establish robust evidence-based dietary recommendations for RA patients. Overall, incorporating dietary strategies with anti-inflammatory properties may offer a valuable addition to conventional RA management and potentially improve patient outcomes and quality of life.

## Introduction

Rheumatoid arthritis (RA) is a systemic and chronic inflammatory autoimmune disease affecting ~1% of the world population (1). RA is usually observed between the ages of 20–45 years and more frequently in women than in men (2) and is characterized by pain, swelling, stiffness and loss of function in the joints. Involvement of small joints such as hand and foot joints is common in the body. This disease seriously affects quality of life with increasing morbidity and mortality (3).

The etiology of RA is considered multifactorial as it involves both genetic and environmental factors. Immune mechanisms such as the production of inflammatory cytokines [tumor necrosis factor-α (TNF-α), interleukin-1 (IL-1), interleukin-6 (IL-6)] and increased cell-mediated immune response are known to play an important role in RA formation (4, 5). Recently, nutritional interventions and dietary patterns have become important to improve RA symptoms as RA patients perceive rapid changes in pain and/or swelling after consumption of certain foods. Adoption and maintenance of an appropriate nutritional plan in these patients contributes to the reduction of the severity of disease symptoms and achievement of remission (6). In general, it has been reported that adequate dietary protein and energy intake and a diet rich in antioxidant vitamins and minerals and omega-3 polyunsaturated fatty acids may prevent tissue damage and suppress the inflammatory process in rheumatologic diseases (7). These nutrients have been shown to be effective in preventing free radical formation and increased cytokine levels (8). The nutritional plan may modulate RA symptoms by influencing the patient's metabolic profile and increasing antioxidant levels, as well as altering the intestinal microflora (9). The Mediterranean Diet and Anti-inflammatory Diet have a positive effect on the course of the disease because it contains all the energy, nutrients and polyphenols required for adequate and balanced nutrition (10).

Although the Mediterranean Diet and Anti-inflammatory diet or dietary inflammatory index, which is part of the Mediterranean diet show promise in managing RA, the variability of individual responses to dietary interventions should be taken into account. There are conflicting results regarding the efficacy of DII and MedDiet in reducing disease activity. It is reported that more research is needed to establish robust evidence-based dietary recommendations for RA patients (11). However, while dietary interventions can complement pharmacologic treatments, they should not replace standard medical care. In addition, the inclusion of dietary strategies with anti-inflammatory properties may contribute to RA management and potentially improve patient outcomes and quality of life.

The aim of this review is to examine the current evidence and controversy on the relationship between DII was shaped as the infrastructure of Mediterranean-style eating habits and MedDiet approaches in the nutritional management of RA and their impact on disease course and symptoms, and to develop recommendations for the future.

## Materials and methods

A literature search was conducted using electronic databases, MEDLINE, Embase, Cochrane Library, CINAHL, Clinical Trials.gov, Scopus, Pubmed, Google academic, ScienceDirect and Web of Science. The reference articles were obtained from databases using the keywords: [RA] and [diet] or [dietary pattern] or [diet quality] or [nutrition] or [dietary inflammatory index] and [MedDiet] and [mechanism] and [pathway].

Searches were performed by the authors and full texts were categorized according to studies published between 2018 and 2025, their availability in English full-text format, and their status as original research, review, traditional review, systematic and meta-analysis, and letter to the editor.

Articles written in languages other than English or published as preprint versions were excluded during the screening process. We also excluded articles that were considered to be of low relevance or irrelevant to our areas of interest, as indicated by their titles and subtitles. Publications deemed appropriate for review were carefully evaluated, full-text review and discussion were conducted where necessary, and some suggestions for the future were presented.

### Effect of Mediterranean diet and dietary inflammatory index (DII) in patients with RA

Although the clinical findings of RA vary according to the stage of the disease and the joints involved, the most prominent findings are symmetrical swelling of the joints and intense pain (12). In addition to these, morning stiffness, fever, fatigue and weakness are other findings of the disease which progress slowly over a few weeks (13). Adoption and maintenance of an appropriate nutritional plan in patients with rheumatoid arthritis contributes to the improvement of the disease, reduction of the symptoms and risk of complications of the disease, and achievement of the patients' remission period and may positively affect the quality of life. Therefore, a nutrition plan is recommended to complement existing treatments (14, 15). Nutrition may have a direct role in disease development through the provision of (anti)-inflammatory food components. In addition, it may have an indirect effect through its effects on BMI, visceral fat accumulation and contributing to the prevention of the development of chronic diseases such as diabetes and CVD as complications (16). Although there is no clear evidence on the effect of dietary plan and treatment on disease activity, various nutrients in the diet may affect disease activity by interacting with the immune system and suppressing inflammation (17). In this context, the dietary plan for RA aims to alleviate inflammation by changing the ratio of ω-6 to ω-3 fatty acids and increasing antioxidants. It is emphasized that reducing arachidonic acid (AA), a ω-6 fatty acid, is particularly important and AA is a precursor of eicosanoids. Eicosanoids are mediators of inflammation and the amount of AA released from the cell membrane can determine the intensity of inflammation (18). It has been reported that various nutrients, phenolic substances, spices such as ginger and turmeric, various vitamins and probiotics control the activity of inflammatory molecules involved in the pathophysiology of RA and thus are successful in slowing down the course of the disease (19). Since nutrients are found in combination in foods, dietary patterns are investigated rather than the effect of a single nutrient on disease. Therefore, the relationship between certain dietary patterns and health is examined instead of considering foods or nutrients alone (20).

### The Mediterranean diet (MedDiet), dietary inflammatory index (DII) and its relationship with RA

**Figure 1:**
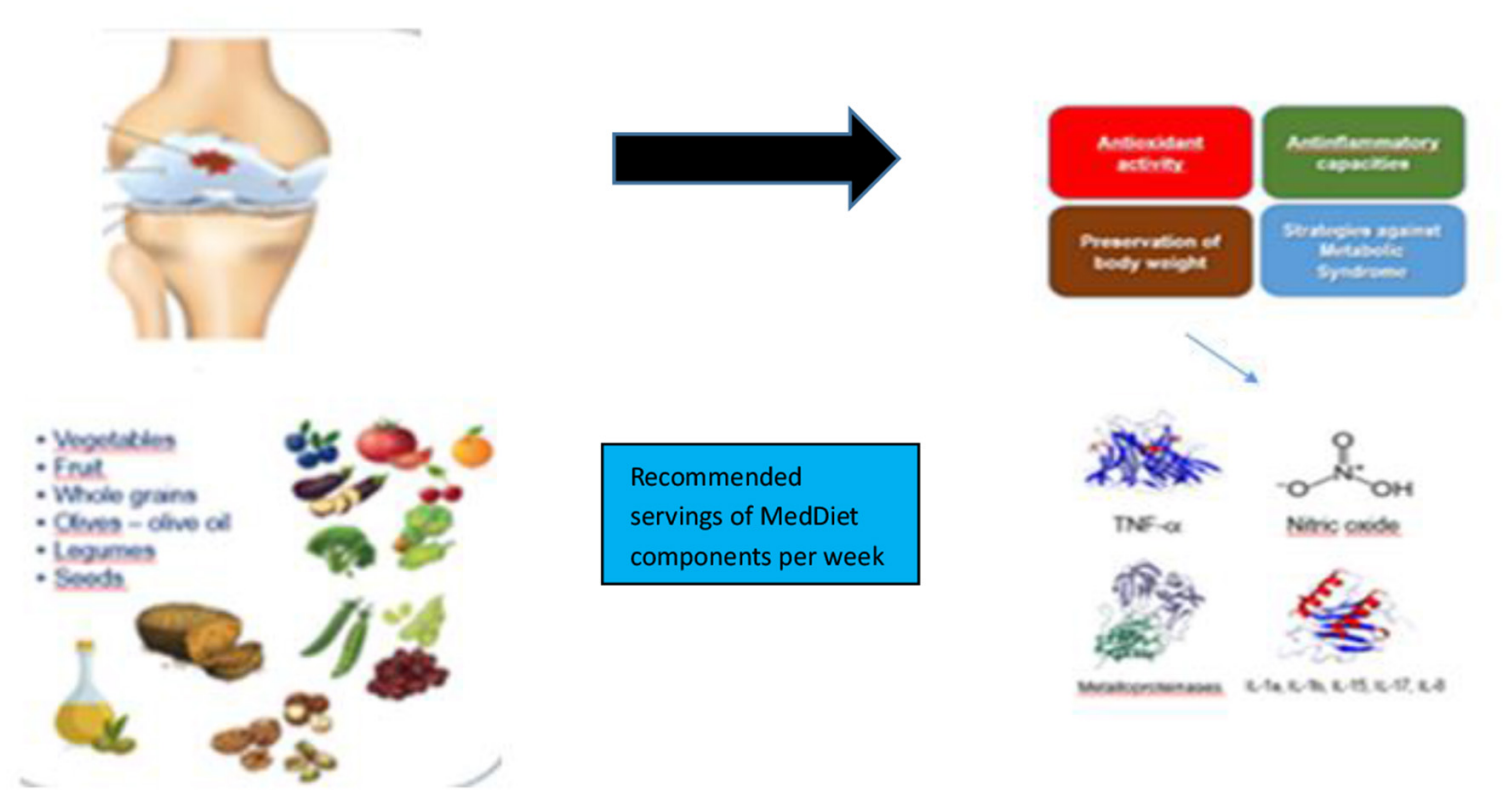
The effect of MedDiet on RA.

**Figure 2:**
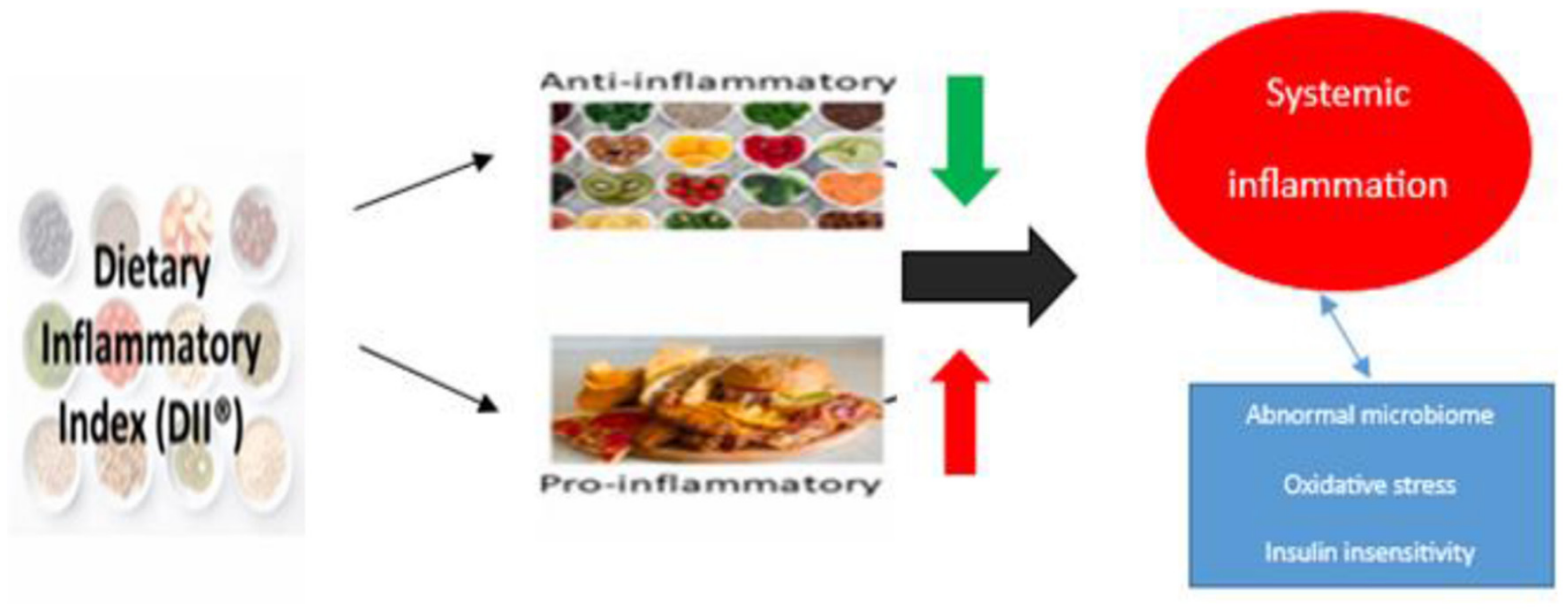
The effect of dietary inflammatory index on RA.

The MedDiet is one of the most widely studied and well-known dietary models worldwide. The traditional MedDiet model is closely related to individuals‘ social behaviors and lifestyles (21). The Mediterranean diet (MedDiet) is increasingly recognized for its potential benefits in the treatment of rheumatoid arthritis (RA), primarily due to its anti-inflammatory and immunomodulatory properties (22). The diet, rich in fruits, vegetables, whole grains, olive oil, and fish, is thought to affect RA through several mechanisms, including modulation of inflammatory pathways, improvement of gut microbiota, and reduction of disease activity scores (23). Collectively, these mechanisms may contribute to alleviating RA symptoms and improving patients' quality of life. The effect of MedDiet and Antiinflammatory DII on RA is shown in Figures 1, 2.

### Effective mechanisms and signaling pathways of MedDiet and dietary inflammatory index on RA

MedDiet can reduce disease symptoms and improve the quality of life of individuals with RA through effective mechanisms such as suppressing inflammation, stimulating the immune system and regulating microbiota (24).

#### Anti-inflammatory effects

The MedDiet has been shown to affect RA-related signaling pathways, primarily through its anti-inflammatory and immunomodulatory effects. Components of the diet, such as omega-3 fatty acids, antioxidants and polyphenols, play an important role in regulating inflammatory pathways central to the pathogenesis of RA. These dietary components can influence the expression of pro-inflammatory genes and the activity of immune cells. Thus, they can potentially reduce disease activity and improve patient outcomes (25). In addition, omega-3 fatty acids, antioxidants and polyphenols in MedDiet content may reduce the inflammatory response in RA patients by down-regulating pro-inflammatory gene expression. MedDiet adherence has been reported to be associated with decreased CRP levels, a marker of inflammation, and reduced disease activity scores (DAS28) in RA patients (26). MedDiet is rich in omega-3 polyunsaturated fatty acids (PUFAs), which regulate the function of lymphocytes and monocytes, key players in the immune response. These fatty acids can inhibit the arachidonic acid cascade by reducing the production of pro-inflammatory eicosanoids (27). The antioxidants in the MedDiet, such as those found in fruits, vegetables and nuts, have powerful anti-inflammatory properties. They may inhibit NF-kB activity, a critical transcription factor in the inflammatory response, and thus reduce inflammation in RA (28). MedDiet is also high in phenolic substances as it contains fruits and vegetables and can reduce oxidative stress. These compounds can scavenge ROS, and because they have several hydroxyl groups, polyphenols can neutralize free radicals by giving them a hydrogen atom, thus polyphenols strengthen the endogenous antioxidant defense system (29).

#### Modulation of the gut microbiota

The MedDiet can influence the composition of the gut microbiota, which plays an important role in immune system regulation. High adherence to the MedDiet has been associated with a healthier gut microbiota profile characterized by a reduction of proinflammatory bacteria such as Prevotella copri. This gut microbiota modulation is thought to contribute to the reduced disease activity observed in RA patients following the MedDiet (30). In addition, MedDiet may also affect the immune system by acting as an epigenetic regulator. It affects the gastrointestinal epithelial barrier and mucosal immune system, which are important in the pathogenesis of autoimmune diseases such as RA. Components such as curcumin and capsaicin found in spices, which are an integral part of the MedDiet, have been shown to regulate oxidative stress and inflammation by blocking NF-kB and cyclooxygenase 2 pathways (31). Among the factors that play a key role in the pathogenesis of RA are the reactions of leukocytes and proinflammatory cytokines such as TNF-a. In addition, cytokines increase the production of CRP by the liver, which is a strong indicator of synovial inflammation (32). In addition, ROS contribute to the development of joint damage in RA. These compounds cause destruction of hyaluronic acid and damage membrane function by oxidation of collagen, proteoglycans, protease inhibitors and membrane fatty acids (33).

#### Improvement in disease activity and quality of life

It reported that RA patients who adhered to the MedDiet experienced significant improvements in disease activity scores, physical function and quality of life. The MEDRA study found that patients on the MedDiet had better physical function and quality of life compared to those following standard dietary guidelines (34). The effect of the MedDiet in reducing RA disease activity is also supported by findings showing a correlation between higher MedDiet adherence scores and lower DAS28 scores. It has been emphasized that MedDiet adherence is associated with a decrease in DAS28 in RA patients, indicating that the diet may positively affect disease activity scores (35).

#### Potential for disease prevention

Longitudinal studies suggest that adherence to the MedDiet may reduce the risk of developing RA. This protective effect is partly mediated by reductions in BMI and CRP levels, emphasizing the role of diet in preventing the onset of RA (36). It has been reported that there is an inverse relationship between the Mediterranean Adherence Score and RA risk and that high adherence to the MedDiet reduces the risk of RA development by 21% compared to low adherence (37). On the contrary, it has also been reported that the Mediterranean diet has no significant protective effect on the risk of RA development (38). In a case-control study, it was reported that MedDiet had no protective effect on the risk of RA development in individuals followed for a period of 7 years before the onset of RA symptoms (39). It has been reported that there is no association between the Mediterranean diet and the risk of RA development. However, it has been emphasized that it can provide an improvement in the clinical symptoms of RA, including disease activity, inflammatory markers and physical function (26).

A diet low in (pro-inflammatory) foods and nutrients such as refined grains, red meat, trans fatty acids, saturated fatty acids, omega-3 fatty acids, monounsaturated fatty acids, antioxidants, phytochemicals, flavonoids, and vitamin D is low in DII, while a diet rich in foods and nutrients such as fruits containing proteolytic enzymes such as papain and bromelain (papaya, mango, pineapple), ginger, turmeric, black pepper, green tea, and legumes is high in DII (anti-inflammatory) (40). These nutrition approach work through several mechanisms, including reducing systemic inflammation, regulating gut microbiota and improving overall nutritional status, which collectively contribute to alleviating RA symptoms (41).

#### Food intake and pathways of inflammation

Anti-inflammatory diets are rich in polyunsaturated fatty acids, polyphenols and antioxidants, which have been shown to reduce inflammation and oxidative stress, important contributors to RA pathology (42). Certain nutrients, such as omega-3 fatty acids found in fish oil, have been associated with reduced production of pro-inflammatory eicosanoids, leading to reduced joint pain and swelling (18). Higher intakes of micronutrients such as vitamin C, niacin and magnesium have been linked to lower disease activity scores in RA patients, suggesting their role in regulating inflammatory responses (43). Increased intake of cereal fiber has been associated with a decreased prevalence of RA, and DII has been shown to mediate this relationship, emphasizing the importance of dietary fiber in the management of RA due to its anti-inflammatory effects (44). While consumption of oily fish is associated with suppression of inflammation and lower disease activity and fatigue in RA patients, consumption of processed meat has been reported to have the opposite effect (45). Monounsaturated and polyunsaturated fatty acids in the anti-inflammatory diet are thought to prevent tissue damage and reduce inflammation (46). Se, an antioxidant mineral, has been reported to have a suppressive effect on inflammation by scavenging ROS and increasing glutathione peroxidase (GPx) enzyme activity (47).

#### Modulation of gut microbiota and metabolism

Dietary interventions can alter the gut microbiome, which in turn affects systemic inflammation. Anti-inflammatory diet has been shown to alter the composition of the gut microbiota and metabolome, which is associated with pain response in RA patients. The presence of certain gut microbes such as Akkermansia, which are enhanced by anti-inflammatory diets, may be linked to the production of short-chain fatty acids with anti-inflammatory properties (48). Furthermore, most dietary fiber is fermented by intestinal bacteria and thus microbial metabolites lead to the production of short-chain fatty acids (SCFAs). SCFAs have been shown to contribute to the gut microbiota (49). Reduced dietary fiber intake and increased fat and sugar intake in Western diets have been shown to contribute to gut microbial dysbiosis by depleting specific bacterial taxa of the gut microbiota. It has been emphasized that microbial dysbiosis in the gut promotes severe immunological dysfunctions that may contribute to the immune imbalance observed in RA (50). In this context, it has been suggested that the onset and severity of clinical arthritis symptoms may be reduced depending on gut microbiota composition, which may be affected by dietary fiber supplementation in RA diseases (51).

#### Dietary patterns and disease activity

An anti-inflammatory diet high in fruits, vegetables, whole grains and healthy fats has been associated with reduced disease activity and improved disease manifestations in RA (52). It shows that adherence to anti-inflammatory dietary patterns can lead to significant improvements in pain, joint swelling and overall quality of life in RA patients (1). Dietary Inflammatory Index (DII) scores show that diets with lower inflammatory potential are associated with reduced disease activity scores in RA patients (53). Omega-3 fatty acids, one of the anti-inflammatory dietary components, have been reported to reduce morning stiffness and the number of tender and swollen joints in patients with RA (54). In a review study, it was reported that 2.1–9.1 g/day omega-3 supplementation reduced the pain level in patients with RA (55). After anti-inflammatory dietary intervention, it was reported that RA had a positive effect on disease activity score and joint findings (such as pain, swelling, stiffness) and CRP level (56). In a case-control study in RA patients, it was reported that the group consuming 2.36 mg/day n3-fatty acid-enriched foods for 10 weeks showed a significant improvement in clinical findings such as disease activity, pain and fatigue compared to the control group (57). While evidence supports the beneficial role of anti-inflammatory diets in managing RA, it is important to consider variability in individual responses to dietary interventions. Factors such as basic dietary habits, genetic predispositions, and current gut microbiota composition may influence the efficacy of dietary changes.

### Signaling pathways for the effects of an dietary inflammatory index on RA

The signaling pathways involved in the effect of an anti-inflammatory diet on RA are complex and multifaceted and involve various molecular and cellular mechanisms. An anti-inflammatory diet rich in nutrients with antioxidant properties has been shown to reduce disease activity and improve symptoms in RA patients. This dietary approach affects several important signaling pathways, including NF-κB, JAK/STAT and the cholinergic anti-inflammatory pathway. These pathways play important roles in regulating inflammation and immune responses in RA (58).

#### NF-κB signaling pathway

NF-κB is a critical transcription factor involved in the regulation of genes responsible for inflammation and immune responses. Inhibition of the NF-κB pathway is seen as a therapeutic target in RA as it can reduce the expression of pro-inflammatory cytokines and other mediators of inflammation. Nutraceuticals and dietary components that inhibit NF-κB activation can potentially alleviate RA symptoms by reducing inflammation (59). Polyphenols in the anti-inflammatory diet have been reported to increase NF-κB phosphorylation and down-regulate AMPK phosphorylation (60). The abnormal NF-κB signaling pathway in RA may cause inflammation. Anti-inflammatory diet significantly decreases RAW 264.7 inflammatory activity, nitric oxide production, PGE2, proinflammatory cytokines (TNF-α, IL-1β and IL-6) and PTGES2 in RAW 264.7 macrophages. This mechanism leads to decreased phosphorylation of NF-κB, a transcription factor that modulates inflammatory proteins including JNK, ERK1/2 and p38, downregulating the MAPK signaling pathway (61). It has also been emphasized that phenolics, one of the anti-inflammatory diet components, significantly reduce the protein expression of COX-2, iNOS and IL-1β and inhibit the PI3K/AKT/NFκB pathway, which is closely linked to RA (62).

#### JAK/STAT signaling pathway

The JAK/STAT pathway is activated by various cytokines, including interleukins and interferons, which are involved in the inflammatory processes of RA. Anti-inflammatory diets can regulate this pathway by affecting the expression of cytokines and their receptors, thereby reducing inflammation and disease activity. Some anti-inflammatory cytokines such as IL-4 and IL-10 also activate the JAK/STAT pathway, emphasizing the complexity of its regulation in RA (63). Flavonoids, a component of the anti-inflammatory diet, are involved in the regulation of Janus kinase (JAK) signal transducer and activator of transcription (STAT) [(Janus kinase (JAK)-signal transducer and activator of transcription (STAT); JAK STAT)] signaling pathways (64). Zinc is thought to play a role in modulation of the NF-kB signaling pathway through A20 zinc finger and OS attenuation, decreasing ROS levels and reducing chronic inflammation by preventing NF-kB activation. A20 up-regulates A20 expression in response to various stimuli such as ROS, TNF-α and IL-1b and has been reported to act as an important inhibitor of NF-kB activation contributing to the down-regulation of inflammation (65).

#### Cholinergic anti-inflammatory pathway (CAP)

The cholinergic anti-inflammatory pathway (CAP) is known as a classic neuroimmune pathway consisting of the vagus nerve, acetylcholine (ACh), the main neurotransmitter of the vagus nerve, and its receptors. This pathway can activate and regulate the activities of immune cells, inhibit cell proliferation and differentiation, and suppress cytokine release, thus playing an anti-inflammatory role (66). This mechanism is associated with acetylcholine's ability to suppress the release of pro-inflammatory cytokines through nAChR activation. Vagus nerve stimulation (VNS) has been shown to activate CAP leading to reduced systemic inflammation and improvement of RA symptoms (67). CAP activates nAChRs to suppress pro-inflammatory cytokine release, a pathway that plays an important role in the modulation of inflammation in RA, In particular, it interacts with α7nAChR to reduce inflammation and improve clinical outcomes (68). The cholinergic anti-inflammatory pathway regulates the proliferation and differentiation activities of various immune cell subsets through peripheral nerve communication with immune cells. This pathway represents a potential target for the treatment of autoimmune diseases such as rheumatoid arthritis, characterized by marked inflammation and decreased vagal tone (69). RA is widely recognized as a disease driven by CD4+ T cells. As an important component of innate immunity, macrophages also contribute significantly to the immune abnormalities associated with RA. Manipulating CAP in immune cells is seen as a viable way to treat RA (70). This is because the cholinergic anti-inflammatory pathway (CAP) has been identified as an important aspect of neuro-immune regulatory feedback and the interaction between acetylcholine and the alpha 7 nicotinic acetylcholine receptor (α7nAChR) underlies this signaling. Consistent with its immunomodulatory functions, α7nAChR is densely expressed by immune cells and CAP activation greatly affects the differentiation and function of α7nAChR-expressing immune cells (71).

#### Cytokine signaling and iNOS regulation

Proinflammatory cytokines such as IL-1, TNF-α and IL-6 can stimulate ROS production through activation of various cellular pathways. Excessive ROS production leads to oxidative stress and cellular damage and both endogenous and exogenous antioxidants can reduce the inflammatory response and ROS production by regulating cytokine production and activity. Antioxidants scavenge ROS and protect cells from oxidative damage, thus alleviating cytokine-induced inflammation and maintaining redox balance (72). Cytokines such as TNF-α, IL-1 and IL-6 are the main mediators of inflammation in RA. Dysregulation of cytokines plays a role in various diseases, especially autoimmune disorders. In RA, abnormal production or signaling of proinflammatory cytokines such as TNF-α and IL-6 contribute to chronic inflammation and tissue damage and are considered good targets for dietary interventions (73, 74). The cytokine signaling network regulating iNOS includes pathways such as IFN and IL-10, which are upregulated in RA synovium, while the TGF-β pathway is downregulated. The transcription factor STAT1 and the iNOS-interacting protein RAC2 are consistently upregulated in RA, indicating that they are involved in the regulation of NO production and chronic inflammation. iNOS regulation by cytokines is crucial in RA as it contributes to inflammation and tissue damage. Anti-inflammatory diets may reduce nitric oxide production and inflammation by affecting cytokine and iNOS expression (75).

#### Dietary impact on inflammatory markers

It has been shown that an anti-inflammatory diet can lead to a reduction in disease activity scores (DAS-28) and improve quality of life in RA patients (76). Nutrients such as iron, vitamin C, niacin and magnesium, which are generally higher in anti-inflammatory diets, have been associated with reduced inflammation and improved clinical outcomes in RA (77). An anti-inflammatory diet for patients with rheumatoid arthritis may be effective in improving the symptoms of the disease by reducing gene expression of markers such as IL-1, IL-6 and TNF-α, which are effective in the inflammation processes of RA (78). It has been emphasized that Se intake improves some disease states in RA patients, such as alleviating pain, reducing the number of tender joints and shortening the duration of morning stiffness (79). In a study, it was shown that there was a weak but significant relationship between DII scores and RA disease activity. DAS-28 scores of patients consuming an anti-inflammatory diet were found to be lower compared to those consuming a proinflammatory diet (80). In a case-control study examining the effect of antioxidant minerals on rheumatoid arthritis, it was observed that the patient group had significantly low intake and low plasma concentration of zinc mineral. As a result, it was suggested that zinc deficiency in rheumatoid arthritis patients decreased the activity of antioxidant enzymes (superoxide dismutase and glutathione peroxidase) and increased OS (81). In a single-blind, randomized, crossover study on patients with RA, after a 1-month washout period, the groups were crossed and intervention and control diets were administered again for 10 weeks. AD (3–4 times/week fish (mainly salmon), 1–2 times/week legumes and potatoes, whole grains, fruits (pomegranate, blueberries), vegetables, low-fat milk, yogurt, oil seeds, probiotic juices and spices) was used as intervention diet. Disease Activity Score 28-Erythrocyte Sedimentation Rate (DAS28-ESR) was found to be lower after the intervention period compared to the control period. However, no significant difference was found between the intervention and control periods in terms of DAS28-ESR (65).

Although anti-inflammatory diet shows promise in the management of RA, it is important to consider the complexity of the disease and individual variability in response to dietary interventions. The interaction between different signaling pathways and the impact of diet on these pathways requires further research to develop comprehensive dietary guidelines for RA patients. In addition, the potential of dietary interventions to complement pharmacological treatments and improve overall quality of life in RA patients emphasizes the need for continued research in this area.

### Some studies on the relationship between MedDiet and dietary inflammatory index with RA

The MedDiet can reduce RA symptoms by suppressing inflammation, altering the lipid profile, increasing antioxidant levels and changing the microflora of the gut (82). The American College of Rheumatology conditionally recommends the MedDiet for RA management because of its potential to improve physical function, reduce joint swelling, and relieve pain (83). In one study, patients with RA were given MedDiet for 2 weeks and significant improvements were found in pain score and health assessment score (84). In another study, it was reported that compliance with MedDiet led to a decrease in disease activity (85). A 12-week randomized study in patients with RA showed a decrease in disease activity, improvement in physical function and increase in vitality as a result of MedDiet intervention (86). In another study, high intake of monounsaturated fatty acids due to MedDiet intervention was found to be a determinant of disease remission in patients with RA (87). In a randomized controlled trial, MedDiet was found to have positive effects on disease activity in RA (88), while in contrast, another study on women with RA found no significant association between MedDiet adherence and RA risk (86). MedDiet has also been associated with lower levels of depression, a common comorbidity in RA patients, due to its anti-inflammatory properties (89). In a randomized clinical trial, patients with RA were divided into MedDiet+dynamic exercise program (DEP), DEP only, MedDiet only, and control group. It was reported that health-related quality of life increased by 15 points in the AD+DEP and DEP groups and increased by 3.5 points in the MedDiet group, while a decrease of 4.6 points was observed in the control group (90). In another study, it was reported that ellagic acid, one of the phenolic acids found in pomegranate, decreased inflammation (91). The anti-inflammatory effects of pomegranate and its products (extract and juice) are reported to be mediated through inhibition of cell signaling pathways, including suppression of cyclo-oxygenase-2 and inducible nitric oxide expression, inhibition of NF-κB activation and inhibition of phosphorylation of MAPK proteins (92). In a case-control study, the intervention group received 2 capsules of 250 mg POMx and the control group received 2 capsules of 250 mg cellulose daily for 8 weeks. Compared to the placebo group, those who received pomegranate extract (POMx) supplementation significantly reduced the DAS28 score, which may be associated with a reduction in the number of swollen and tender joints, pain intensity and ESR levels. It was also reported that POMx reduced HAQ score and morning stiffness and increased GPx concentrations (1). In another study, it was suggested that gut microbiota plays a role in the pathogenesis of RA. It was found that POMx could change the gut microbiota and lead to health benefits (93). Despite positive findings, some studies have reported that there is no significant relationship between MedDiet and RA disease activity. One study found no significant difference in disease activity between different levels of MedDiet adherence (94). Another study indicated that MedDiet may have a weak but significant effect in controlling disease activity, but the evidence was not strong enough to provide specific dietary recommendations for RA management (86). There is further evidence of the effects of MedDiet on RA outcomes. In a 3-month randomized controlled trial in RA patients, a MedDiet-based intervention improved markers of disease activity, including CRP and ESR, and the MedDiet group showed significant improvements in physical function and health-related quality of life compared to the control group (95). Another randomized controlled trial, the MEDRA study, found greater improvements in physical function and quality of life in RA patients on the MedDiet (96). MedDiet may also play a role in primary prevention of RA. A prospective cohort study found that higher adherence to the MedDiet was associated with a reduced risk of RA, especially among smokers. This suggests that the MedDiet may attenuate the proinflammatory effects of smoking, a known risk factor for RA (97). The protective effects of the MedDiet on RA risk may be mediated through its anti-inflammatory properties. MedDiet components such as olive oil, fish and antioxidants have been shown to regulate inflammatory pathways and reduce the production of proinflammatory cytokines, which are central to RA pathogenesis (41).

A study using NHANES data (1999–2018) found that higher DII levels were associated with an increased risk of anemia in RA patients, suggesting that dietary inflammation may exacerbate RA-related complications (98). A case-control study found that individuals with the highest DII scores, indicating a more proinflammatory diet, had more than three times the risk of developing RA compared to those with the lowest scores (55). A study analyzing data from NHANES 2003–2018 found that adherence to a healthy and anti-inflammatory diet characterized by a low DII score was associated with reduced all-cause mortality in RA patients. This suggests that dietary changes may be a strategy to improve long-term health outcomes in RA (99). High DII scores were positively associated with SII and NLR in RA patients. It has been stated that this may indicate that a pro-inflammatory diet may exacerbate systemic inflammation (65). In a cross-sectional study, it was determined that CRP and disease activity scores were significantly lower in patients with RA who consumed fish twice a week or more frequently than those who consumed fish never or once a month or less (100). In a study examining RA risk with spices, another important component of an anti-inflammatory diet, it was reported that consumption of 1.5 g/day ginger for 3 months reduced the risk of disease activity and CRP level compared to the control group (101). In another study, it was reported that consumption of 2 g/day cinnamon for 2 months significantly improved disease activity scores and reduced the number of swollen joints compared to the placebo group (102). Another study found that consumption of 100 mg/day saffron for 3 months improved disease activity and joint pain compared to the control group (103). B6, another antioxidant vitamin, is associated with the risk of rheumatoid arthritis (104). In a study, circulating PLP was found to be inversely correlated with C-reactive protein (CRP), erythrocyte sedimentation rate, pain level, morning stiffness and disability score in RA patients, and low vitamin B6 intake was associated with high inflammation (105). In a meta-analysis study, RA patients were shown to have low serum Zn concentrations compared to healthy controls (106). In RA patients, low plasma zinc levels can be attributed to a change in zinc homeostasis that triggers an acute inflammatory response and removes zinc from plasma to the liver. This process is mediated by IL-1b through nitric oxide induction and IL-6 upregulating ZIP14 antibody in the liver, which can cause zinc sequestration and redistribution, leading to an inflammatory response (107). In a study by Rajaee et al. (108) a statistically significant inverse relationship was observed between serum Zn levels and DAS28 score. In another study, it was reported that the activity of SOD and GPx enzymes, which are important for the antioxidant defense system, was significantly lower in the RA group compared to the control group (109). It has been observed that consumption of dietary fiber, another important component of the anti-inflammatory diet, increases the dietary DDI index and shows a significant inverse correlation with the incidence of RA (110). In a study, it was found that RA patients who were fed a diet rich in dietary fiber for 3 years had a significant decrease in DAS28 scores and an increase in quality of life assessed by HAQ and SF-36 health questionnaires compared to control patients (111).

Some studies examining the effects of Mediterranean Type and Anti-Inflammatory Nutritional Therapy on the risk of RA development and disease symptoms are presented in Table 1.

**Table 1:** Some studies on Mediterranean and anti-inflammatory dietary interventions in the treatment of RA.

**Reference**	**Type of study**	**MedDiet intervention**	**Antiinflamatory Diet intervention**	**Outcomes**
Veselinovic et al. (51)	Prospective, randomized	60 patients with RA were divided into 3 groups for 12 weeks; Group receiving fish oil: FO providing 1.5 g DHA/d, 1.0 g EPA/d, and 500 mg/d of other n-3 PUFAs Group receiving fish oil + evening primrose oil: EPO providing 2.6 g EPA/d, 1.9 g LA/d, and 2.3 g GLA/d Group receiving no supplementation.	–	DAS 28 score, number of tender joints VAS score decreased significantly after supplementation in groups I and II
Javad et al. (113)	Randomized controlled		RA patients were given 500 mg quercetin daily for 8 weeks and the control group was given placebo	DAS-28 and HAQ scores, TNF- α level and number of tender joints decreased significantly
Thimóteo et al. (114)	Randomized controlled		Cranberry group (*n* = 23) consumed 500 ml/day cranberry juice for 90 days, control group (n=18) consumed placebo	There was a decrease in DAS-28 and anti-CCP values. In addition, HDL level increased in the cranberry group
Sparks et al. (115)	Randomized controlled	Frequent fish consumption (2 times/week Infrequent fish consumption (none or < 1 serving/month)		No significant protective association was found between seropositive RA and fish intake
Amalraj et al. (116)	Randomized double-blind placebo-controlled		250 mg and 500 mg of curcumin extract and placebo were given twice daily for 90 days.	Significant differences were observed in clinical symptoms, ESR, CRP and RF levels in the groups receiving curcumin compared to the placebo group
Javadi et al. (117)	Randomized double-blind placebo-controlled		30 of 65 patients with RA were given curcumin nanomcelle (40 mg) 3 times daily for 12 weeks, while 35 patients received placebo.	No significant difference was found between the groups in terms of DAS-28 score, number of swollen and tender joints
				
Badsha et al. (17)	Randomized	51 patients with RA followed a Mediterranean diet for 12 weeks		Decreased disease activity and improved physical function
Athanassiou et al. (20)	Single-blind, randomized controlled	RA patients were given a Mediterranean diet for the first 7–10 days and a vegan diet for 3.5 months for 1 year.		Reduced number of tender and swollen joints and improved health assessment questionnaire score
Rondanelli et al. (118)	Observational		In a study of 40 women with RA, daily supplements of 50 μg selenium, 8 mg zinc, 125 mg vitamin C and 40 mg vitamin E were given	Improved clinical outcomes and alleviated oxidative stress in RA
Guagnano et al. (119)	Intervation	Patients with RA were administered MedDiet 3 months deprived of meat gluten and dairy products	.	Improvement in VAS and SF-36 scores was observed in the intervention group compared to the control group
Jiang et al. (120)	Randomized-Intervation		Patients with RA are included in a high-fiber diet.	Reported reduction in pain, fatigue and inflammation markers
Mukherjee et al. (121)	Randomized-Intervation	50 patients with RA were assigned to a Mediterranean diet for 10 weeks and a control group.		Patients in the intervention group had lower disease activity and CRP, RF and sedimentation values
Gonçalves et al. (122)	Prospective		The effect of phenolic compounds in the diet of RA patients on the symptoms of the disease was examined.	It was emphasized that compounds such as quercetin, catechin, curcumin suppress inflammation
Bekar et al. (123)	Prospective		The relationship between the level of adherence to the Mediterranean diet and serum antioxidant shutdown in 35 women with RA and 35 healthy women was analyzed	Compliance with the Mediterranean diet and intake of protein fiber, EPA, vitamin A, Fe, Zn and total antioxidants were lower in the RA group compared to the control group. Serum total antioxidant status was lower and oxidative stress index was higher in the RA group.
Renard et al. (124)	Randomized-controlled	392 individuals with RA were randomly assigned to Mediterranean diet and control group.		51% of patients on the Mediterranean diet experienced a reduction in pain
Dhankhar et al. (125)	Prospective	The intake of phenolic compounds and their effect on disease findings in individuals with RA on a Mediterranean diet		Phenolics kaempferol, luteolin, genistein and daidzin reduced joint swelling
Termine et al. (126)	Prospective	Important components of the Mediterranean diet MUFA (especially olive oil) ve PUFA(especially fish oil) observed in studies and their effect on rheumatoid arthritis outcomes in patients		Consumption of fish at least twice per week and Higher consumption of olive oil show that positive impact
Meng et al. (127)	Prospective		To investigate the association between dietary antioxidant index (DI) and RA risk, 26501 individuals with RA were studied.	Suggested that higher dietary antioxidant intake is associated with lower incidence of RA

## Conclusions

Rheumatoid arthritis (RA) is an autoimmune, inflammatory, progressive disease that results in joint damage and has an unknown cause. Although nutritional therapy affects the risk of developing RA, it can improve clinical and biochemical symptoms (such as ERS, CRP) such as disease activity, morning stiffness, joint pain, and fatigue in individuals diagnosed with RA (112).

While The Mediterranean diet and its anti-inflammatory properties show promise in managing RA, it is important to consider that dietary interventions alone may not be sufficient for all patients. The complexity and multifactorial nature of RA requires a comprehensive approach that includes lifestyle modifications as well as pharmacologic therapies. Many studies are observational or have small sample sizes. Therefore, findings may not be generalizable to all populations. In addition, the lack of standardized tools to assess adherence to the MedDiet and DII across studies may contribute to inconsistencies in results. Some studies have suggested significant improvements in disease activity and quality of life, while others have found no significant effect.

Mediterranean diet and anti-inflammatory eating habits, another aspect of the Mediterranean diet may be effective in reducing the risk and symptoms of the disease by reducing inflammatory markers or increasing antioxidant defenses. Since drug therapy is costly and causes side effects in patients, dietary therapy should be supported. In addition, patients' dietary habits, nutrient and nutrient intakes, biochemical findings, physiological findings, physical activity level and quality of life level should be evaluated in detail and individualized nutrition plans may be beneficial in RA patients (128). In addition, although beneficial effects of MedDiet and anti-inflammatory diet have been shown, individual responses may vary and further research is needed to fully understand the mechanisms and optimize dietary recommendations for RA patients.

## Limitations

While this review provides an insight into the potential role of nutrition in the management of this complex condition, it is also important to recognize certain limitations of the research. First of all, there are very few studies investigating the direct effects of dietary patterns in the treatment of RA. While the majority of studies show a positive effect on RA, it is difficult to determine precisely which dietary intervention is effective on specific patient subgroups or specific symptoms. There is also considerable heterogeneity between different studies, as study designs, methodologies, and number of patients vary greatly. It is therefore difficult to generalize from the results. It is difficult to establish a link between different dietary dietary models and RA, which address hormonal, genetic, immune and environmental factors related to the development of the disease, primarily because dietary model studies in humans unfortunately do not delve deeply into these issues. Furthermore, experimental studies are mostly *in vivo, in vitro* and *in vitro* studies and human studies are scarce. Therefore, it is difficult to generalize the results to the general population and this is a limiting factor of this study.

## Future perspective

There are not enough well-designed randomized controlled trials in the scientific literature to assess the short-term and long-term impact of dietary plans on the onset, progression or treatment of RA. There are not many studies examining the effect of different dietary interventions on RA. Future research should focus on improving the bioavailability of dietary patterns in the treatment of RA and during attack periods. In addition, larger and longer clinical trials are needed to identify well-defined endpoints, to clarify the patients who will benefit from dietary intervention, and to fill the gaps in the literature on disease dietary modifications and their use in the clinic. This review is promising in terms of bridging the gap between nutrition and RA, raising awareness, and the relevance and applicability of the observed results.
